# Synthesis of Chalcones: An Improved High-Yield and Substituent-Independent Protocol for an Old Structure

**DOI:** 10.3390/molecules28227576

**Published:** 2023-11-14

**Authors:** Ana Donaire-Arias, Martin L. Poulsen, Jaime Ramón-Costa, Ana Maria Montagut, Roger Estrada-Tejedor, José I. Borrell

**Affiliations:** Grup de Química Farmacèutica, IQS School of Engineering, Universitat Ramon Llull, Via Augusta 390, E-08017 Barcelona, Spain; anaariasd@iqs.url.edu (A.D.-A.); martinpoulsen@iqs.url.edu (M.L.P.); jaimeramonc@iqs.url.edu (J.R.-C.); anamaria.montagut1@gmail.com (A.M.M.); roger.estrada@iqs.url.edu (R.E.-T.)

**Keywords:** chalcones, aldol condensation, Wittig reaction

## Abstract

Chalcones are a type of molecule that can be considered as easily synthesizable through aldol condensation or that can be readily purchased from habitual commercial vendors. However, on reviewing the literature, one realizes that there are no standard procedures for such aldol condensations, that there exists a wide range of alternative methods for the aldol condensation (indicating that such a condensation is not always simple), and that, in many cases, low yields are obtained that involve purifications by recrystallization or column chromatography. To develop a robust standard protocol independent of the nature of the substituents present on the acetophenone or the benzaldehyde involved in the aldol condensation leading to the chalcone, we made a comparison between an aldol condensation in KOH/EtOH and a Wittig reaction between the corresponding ylide and benzaldehyde in water. We describe an improved procedure for the Wittig reaction and a protocol for the elimination of the Ph_3_P=O byproduct (and the excess of ylide used) by filtration of the crude reaction product through a silica gel plug. We thus demonstrate that such an improved procedure can be a general method for the synthesis of chalcones in high yield and excellent purity and is clearly an improvement on the classical aldol condensation.

## 1. Introduction

**Scheme 1:**
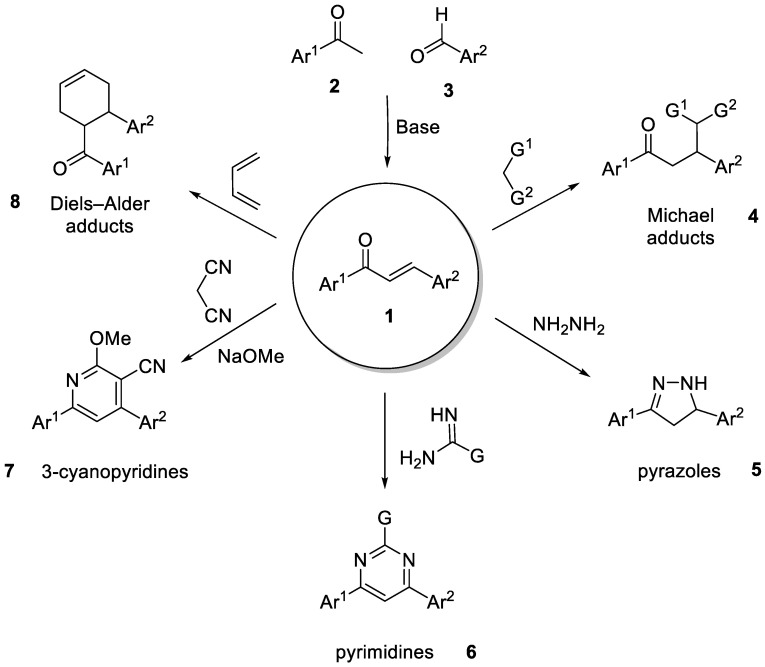
Synthesis of chalcones **1** from arylmethylketones **2** and aromatic aldehydes **3**, with some of their uses in organic synthesis.

Since the synthesis of chalcone (**1**{*1,1*}, Ar^1^ = Ar^2^ = Ph, Scheme 1) by Claisen and Claperède in 1881 [1] from acetophenone (**2**{*1*}, Ar^1^ = Ph) and benzaldehyde (**3**{*1*}, Ar^2^ = Ph), such 1,3-diaryl substituted 2-propen-1-ones have attracted the interest of organic chemists as more than 48,000 compounds **1** included in SciFinder [2] (only considering those presenting phenyl or phenyl fused rings at Ar^1^ and Ar^2^) and numerous reviews [3,4,5] (more than 20 on the synthesis and applications of chalcones) clearly show.

Chalcones **1** have been used as starting products for the synthesis of Michael adducts **4** (Scheme 1), with more than 6800 reactions in SciFinder, using active methylene compounds such as dialkyl malonates, alkyl cyanoacetates, malononitrile, or nitroalkanes including, more recently, enantioselective versions of such reactions [6,7,8]. Compounds **1** have also been used as starting compounds for the synthesis of heterocyclic compounds (Scheme 1) such as pyrazoles **5**, upon reaction with hydrazine or substituted hydrazines [9,10,11]; pyrimidines **6** [12] by reaction with urea, thiourea or guanidines (more than 3300 reactions in SciFinder) [13,14,15,16]; or 3-cyanopyridines **7** by the Michael addition of malononitrile followed by an intramolecular cyclization [17]. Diels–Alder adducts **8** (Scheme 1) have also been obtained from chalcones **1** and dienes (more than 2000 compounds obtained in this way) [18,19,20] and, finally, the stereoselective epoxidation of chalcone (**1**, Ar^1^ = Ar^2^ = Ph) in the presence of poly[[(*S*)-alanine] was developed at our institute by Julià and Colonna in 1980 [21].

Although several methodologies have been described so far for the synthesis of chalcones **1** [4], the most widely used method is still the condensation in basic medium (usually NaOH in EtOH) of an arylmethylketone **2** and an aromatic aldehyde **3** (Scheme 1). In most cases, yields are high but depending on the substituents present in each reagent the conversions may not be so satisfactory and the reaction times can become extensive with column chromatography or recrystallization required to afford the pure chalcone **1**. In some cases, microwave irradiation [22,23] has been used to increase the yield and reduce the reaction time, but such methodologies are not easily scalable due to the nature of the technique and often they are carried out without solvent.

As a part of our ongoing research in the field of MAP kinase-interacting serine/threonine protein kinase 1 (MNK1) inhibitors [24], we needed a rapid and reliable synthesis of chalcones **1**, so we decided, on the one hand, to revise the possible limitations of the Claisen condensation for the synthesis of compounds **1** depending on the nature of the substituents present in the arylmethylketone **2** and aromatic aldehyde **3** and, on the other hand, to determine a general methodology not limited by the nature of such substituents.

**Scheme 2:**
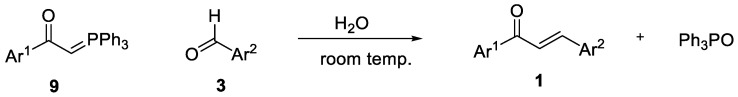
Synthesis of chalcones **1** from stabilized ylides **9** and aromatic aldehydes **3** in H_2_O.

For this second objective, we were highly interested in the Wittig methodology proposed by Dambacher et al. [25] in which an aromatic aldehyde **3** is reacted with a stabilized ylide **9** in water at room temperature to afford the corresponding chalcone **1** in high yields (Scheme 2). Such methodology is quite attractive because organic solvents are not used but it has not received much attention. This is probably because in the reaction triphenylphosphine oxide (Ph_3_P=O) is formed, a byproduct which is always difficult to eliminate—in fact in the aforementioned paper the authors describe the use of flash chromatography employing silica gel 60 Å to purify the products.

Consequently, we decided to try to improve the protocol, and the present paper deals with the results obtained in the study.

## 2. Results and Discussion

As mentioned the Wittig protocol described by Dambacher et al. [25] was selected for the synthesis of chalcone **1**{*1,1*} (Ar^1^ = Ar^2^ = Ph) and its derivatives due to the simplicity of the procedure, the use of water as a solvent, and the potential scalability. After performing the reaction at room temp., as described in the paper, using the ylide **9**{*1*} (Ar^1^ = Ph) and benzaldehyde **3**{*1*} (Ar^2^ = Ph) (Scheme 2), only 16% yield of chalcone **1**{*1,1*} (Ar^1^ = Ar^2^ = Ph) was obtained with standard magnetic stirring—the yield was determined by using ^1^H-NMR since the product was not isolated. However, we noticed that the use of a magnetic stirrer produced aggregation of the initial reactants impeding proper homogenization. To solve this problem a Sonicator bath was used, thus improving the yield to 56%, also determined by NMR. The Sonicator allowed the initial reactants to be better homogenized but mainly in the solid state, therefore, increasing the solubility of the compounds and facilitating the reaction. The mixture was heated at reflux temperature with conventional stirring, ensuring complete solubility, and enabling the yield of chalcone **1**{*1,1*} (Ar^1^ = Ar^2^ = Ph) to increase up to 100% (also determined by NMR) in only 10 min of reaction time.

**Scheme 3:**
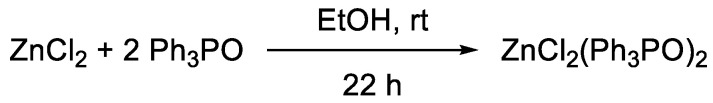
Conditions described by Batesky et al. [26] for the removal of triphenylphosphine oxide by reacting it with zinc chloride.

The main drawback of the Wittig reaction is the formation of triphenylphosphine oxide (Ph_3_P=O), a product that is very difficult to remove without using column chromatography which normally reduces the final isolated yield of **1**{*1,1*}. Batesky et al. [26] described a method for its removal by using zinc chloride (Scheme 3). When we tested this method, unfortunately some triphenylphosphine oxide still remained in the isolated compound **1**{*1,1*}.

During these experiments, we observed that triphenylphosphine oxide was totally retained when a TLC with CH_2_Cl_2_ (DCM) was carried out. Consequently, we used chromatographical filtration on a silica gel plug with the named solvent to remove this byproduct affording chalcone **1**{*1,1*} (Ar^1^ = Ar^2^ = Ph) of very high purity and in quantitative yield. We were able to modify the method to avoid the use of the chlorinated solvent by using a 75:25 mixture of cyclohexane:AcOEt to afford a “greener” and more industrially friendly protocol. Since the reaction is performed in water and the purification method uses organic solvents, a liquid–liquid extraction and a subsequent solvent evaporation to concentrate the product are necessary.

Although the above Wittig protocol is industrially friendly, we decided to test a variation using an organic solvent instead of water. Consequently, we tested THF and 1,4-dioxane as solvents and in both cases solubility was maintained but the reaction time was increased from 10 min to overnight with 1,4-dioxane and 56 h with THF. These results also proved the Dambacher et al. [25] statement on the reduction in reaction time due to the nature of water as solvent. With the use of the mentioned organic solvents, no extraction was necessary, and simple concentration was enough to proceed with the elimination of Ph_3_P=O upon filtration through silica. However, we decided to continue with the reaction in water.

**Scheme 4:**
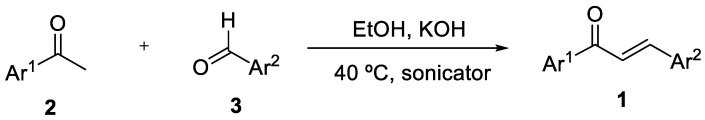
Conditions for the aldol condensation, adapted from Ganesan et al. [27] and Jin et al. [28].

Having to hand a procedure that seems to be easy and robust, we decided to test it with a variety of stabilized ylides **9**{*x*} and benzaldehydes **3**{*y*} to synthesize a wide range of chalcones **1**{*x,y*} having different types of substituents at the ylide **9**{*x*} and the benzaldehyde **3**{*y*}. For comparison purposes, we also obtained the same chalcones **1**{*x,y*} by using the usual methodology for their synthesis, an aldol condensation between an acetophenone **2**{*x*} and a benzaldehyde **3**{*y*} using KOH/EtOH at 40 °C in a Sonicator—the protocol used previously in our group, adapting it from the methodologies described by Ganesan et al. [27] and Jin et al. [28] (Scheme 4).

The two reactions, aldol condensation and Wittig, were performed and compared to prove the effectivity of the Wittig conditions as well as the purification method described above. To carry out such a comparison, the final chalcones **1**{*x,y*} were isolated by rotary evaporation of the reaction solution to avoid material loss. In the case of the Wittig protocol, a 1.5:1.0 molar ratio of the ylide **9**{*x*} with respect to the benzaldehyde **3**{*y*} was used because such an excess was easily removed during the silica gel filtration. The use of an excess of the corresponding acetophenone **2**{*x*} was not possible due to the secondary reactions that occur during the aldol condensation and so an equimolar amount was used.

First, the effect of a *para* substitution on the aldehyde moiety **3**{*y*} was examined, using both electron-donating and electron-withdrawing groups. In this case, no steric interference would be expected in any of the reactions. A search carried out in SciFinder showed more than 200 aldol condensations described, at mg or g scale using NaOH in EtOH, for which the yields are described. Only in almost 39% of the cases were the yields higher than 70%, and only in 42 reactions yields were they between 90–100%.

The results obtained using both protocols are summarized in Table 1.

Comparing both procedures, when electron-withdrawing groups were present at the *para* position of the benzaldehyde **3**{*y*} both the aldol and Wittig reactions performed similarly with very high yields and similar reaction times. Although in all cases the conversion using the Wittig protocol was 100% by NMR, isolated yields were slightly lower due to the silica plug filtration.

**Figure 1:**
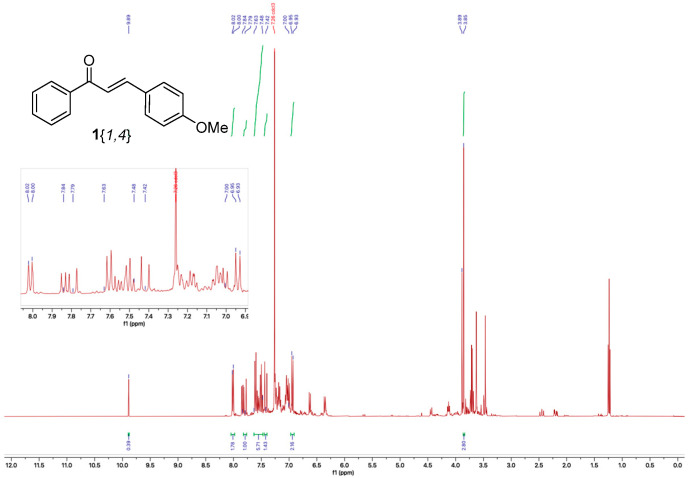
^1^H-NMR spectrum of the crude chalcone **1**{*1,4*} obtained in the aldol condensation of acetophenone **2**{*1*} and the *p*-methoxy substituted benzaldehyde **3**{*4*}.

When an electron-donating group is present at the *para* position of the benzaldehyde **3**{*y*} the differences in the performances of both protocols were remarkable. Although the aldol condensation was faster in all the tested cases, the crude materials obtained needed purification by recrystallization and the highest isolated yield was 74%. As an example, Figure 1 corresponds to the ^1^H-NMR spectrum of the crude material obtained in the aldol condensation of acetophenone **2**{*1*} and the *p*-methoxy substituted benzaldehyde **3**{*4*}, which clearly shows a mixture of compounds, including the desired chalcone **1**{*1,4*}, and remaining benzaldehyde together with residues of the EtOH used in the condensation.

**Figure 2:**
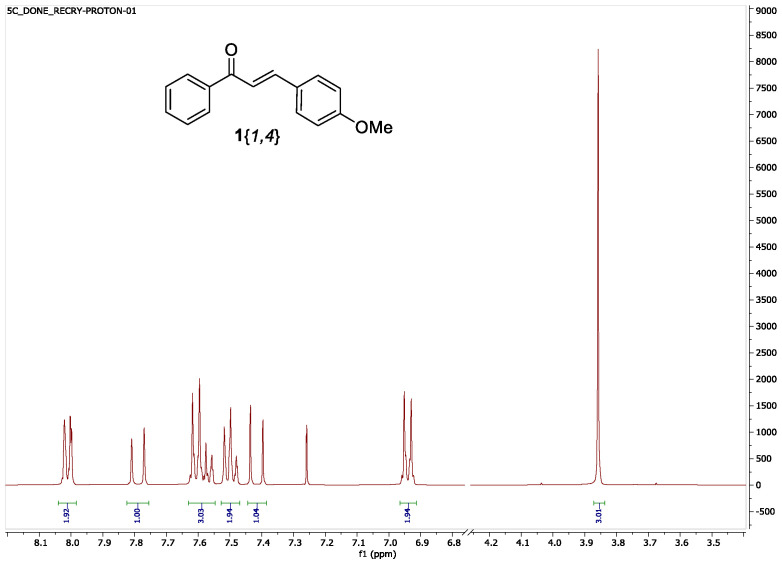
^1^H-NMR spectrum of the crude chalcone **1**{*1,4*} obtained by using the Wittig protocol after Ph_3_P=O was eliminated by filtration through the silica plug.

Although the Wittig protocol needs longer reaction times, the products obtained have a higher purity—in fact in the spectra only the corresponding chalcone and residual dichloromethane are present—and isolated yields increased up to 91–96% (conversion was also 100% by ^1^H-NMR). The Wittig protocol is, therefore, a much better approach when an electron-donating group is present at the *para* position of the benzaldehyde **3**{*y*} since the final products are obtained with higher purity and better yields. Figure 2 shows the ^1^H-NMR spectrum of chalcone **1**{*1,4*}, obtained upon filtration through the silica plug to remove the triphenylphosphine oxide (Ph_3_P=O), showing the high purity of this compound.

The differences can be explained by the nature of the groups present at the *para* position of benzaldehydes **3**{*y*}. The presence of an electron-donating group increases the electron density in the aldehyde group of **3**{*y*} (the electrophile) making the attack of the nucleophile (the enolate of acetophenone **2**{*1*} or ylide **9**{*1*}) less favorable. It is important to consider that, contrary to the Wittig reaction, the aldol condensation is reversible and prone to give secondary reactions. Making the electrophile less reactive increases not only the reaction time but also the impurities present. In the case of the Wittig reaction, although also suffering an electronic effect, the major impact is on the reaction time but not on the yield or the purity of the final compound, thus making it a better and easier option for the synthesis of chalcones, especially with donor groups at the benzaldehyde moiety.

The case of *p*-fluorobenzaldehyde **3**{*5*} deserves special mention. While the Wittig protocol performs quite well and the corresponding chalcone **1**{*1,5*} is obtained in high yield and purity, we observed that the aldol condensation carried out at 40 °C in the presence of KOH/EtOH affords a complex mixture in which two CHO protons are detected in the ^1^H-NMR spectrum together with a triplet and a quadruplet corresponding to an extra ethoxy group. A detailed analysis of the spectrum led us to conclude that the fluorine atom was partially substituted by an ethoxy group, either on the final chalcone or on the starting aldehyde **3**{*5*} as the presence of two different benzaldehydes suggests. This type of substitution has not been previously described for such a benzaldehyde as part of an aldol condensation, but a similar reaction can be found in the literature when **3**{*5*} is treated with Triton-B (benzyltrimethylammonium hydroxide) in methanol at room temp. [29].

Consequently, in this case, it was necessary to change the reaction conditions and carry out the reaction at room temperature, using reaction conditions similar to those described in the literature [30], to obtain a reasonable yield. In any case, the corresponding Wittig reaction between **3**{*5*} and the corresponding ylide **9**{*1*} afforded the chalcone **1**{*1,5*} in 95% yield and high purity, once more showing the better performance of this protocol.

To continue to compare both reactions, steric effects were considered by using benzaldehydes **3**{*y*} with substituents at the *ortho* position. In this case, a search in SciFinder showed a total of 112 aldol condensations (practically half the cases of those with a benzaldehyde with a substituent at the *para* position) carried out at mg or g scale using NaOH in EtOH for which the yields are described. Only in 13 of the reactions (around 12%) were yields in the range of 90–100% showing the effect of steric hindrance on the aldol condensation. We selected three *ortho*-substituted benzaldehydes **3**{*10*–*12*} to test the differences between the aldol condensation and Wittig protocols. The results are summarized in Table 2.

In this case, the electronic effects of the substituents seem not to be so important as in the case of the *para*-substituted benzaldehydes, as electron-donating and electron-withdrawing groups performed similarly. However, the bigger the substituent at the *ortho* position, the longer the reaction time both for the Wittig and aldol condensation. Furthermore, while the isolated yields of the Wittig protocol are in the upper range with conversions around 100% and very high purities determined by ^1^H-NMR, the aldol condensations gave highly colored crude materials still containing 5–10% of the starting materials and requiring careful purifications either by crystallization or column chromatography to afford yields in the medium range.

It is true, that for some of these cases, very high yields have been described in the literature for the aldol condensation (as in the case of the *o*-bromo benzaldehyde **3**{*12*}, G = *o*-Br), but it is also true that very specific reaction conditions have to be used (for instance very long reaction times, high concentration of base, room temp., etc.) which is far from a general procedure. Once more, the Wittig protocol was shown to be a more general and reliable procedure that affords higher yields with purities not being influenced by the initial reagents.

Our last comparison was carried out with benzaldehyde **3**{*1*} and acetophenones **2**{*x*} or ylides **9**{*x*} bearing a substituent at the *para* position of the ring. A search carried out in SciFinder gave a total of 159 aldol condensations of that kind (at mg or g scale using NaOH in EtOH and for which yields are described) and only 54 with acetophenones bearing a substituent at the *ortho* position. In those cases, yields in the range of 90–100% were only achieved in 18 (11%) and 10 (19%) of the condensations with substituents at the *para* and *ortho* positions of the acetophenone **2**, respectively.

However, in this case, the use of the corresponding ylides **9** faces a major problem. Although in SciFinder there are 23 ylides **9**{*x*} commercially available with a wide range of possible substituents at the *para* position, in practice, only the ylide corresponding to acetophenone **9**{*1*} is commercially available from conventional vendors and the others are marketed in mg quantities by companies that obtain them specifically for the customer who requests them. Consequently, to use the Wittig protocol, it is necessary to obtain the required ylide **9**{*x*}.

**Scheme 5:**
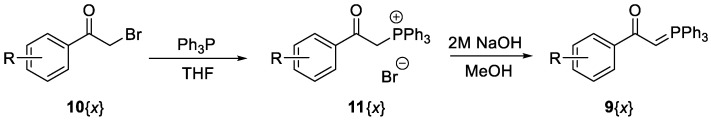
Synthesis of ylides **9**{*x*} from 2-bromoacetophenones **11**{*x*}.

Such ylides **9**{*x*} can be obtained in two steps starting from the corresponding substituted 2-bromoacetophenone **10**{*x*} treated with an equimolar amount of PPh_3_ in anhydrous THF under argon to afford the corresponding substituted (2-oxo-2-phenylethyl)triphenylphosphonium bromide **11**{*x*} in almost quantitative yield. The subsequent treatment of **11**{*x*} with aqueous 2M NaOH in MeOH affords the final ylide **9**{*x*} in a very high yield (Scheme 5). We used this protocol to obtain the ylides **9**{*2*} (R = *p*-Me) [31] and **9**{*3*} (R = *p*-NO_2_) [32].

The results obtained in the synthesis of the corresponding chalcones are included in Table 3.

As can be seen, the performance of the Wittig protocol is once again superior to the classical aldol condensation. Although we tested this protocol and the filtration of the crude reaction product as a means to eliminate the Ph_3_P=O byproduct (and the excess of **9**{*x*} used), in a reduced but significant number of cases, which include electron-donating or electron-withdrawing groups both at the benzaldehyde **3**{*y*} and acetophenone **2**{*x*} (or ylide **9**{*x*}) moieties, the results clearly show that the Wittig protocol is more robust than the classical aldol condensation and almost independent of the nature and position of the substituents present at both reagents participating in the reaction.

## 3. Conclusions

In this work, we compared two different protocols for the synthesis of chalcones **1**{*x,y*}: the aldol condensation between acetophenones **2**{*x*} and benzaldehydes **3**{*y*} in KOH/EtOH at 40 °C in a Sonicator, and the Wittig reaction between ylides **9**{*x*} and the same benzaldehydes in boiling water.

The results obtained for the aldol condensations clearly showed that this reaction only affords excellent results when an electron-withdrawing group is present at the benzaldehyde ring **3**{*y*}. In the rest of the cases, dark-colored crude materials showing several compounds or even unreacted benzaldehyde are obtained. In these cases, recrystallization from EtOH or purification by column chromatography is required to obtain the pure chalcone while paying the price of a significantly reduced yield. For some of the tested chalcones we found in the literature reactions in which the isolated yield is higher than the ones obtained in this work but a careful examination of the reaction conditions showed that they cannot be considered as a standard protocol but require fine-tuning for specific compound **1**{*x,y*}.

On the contrary, the Wittig protocol tested based on the work of Dambacher et al. [25] afforded, in our hands, total conversions for all tested benzaldehydes **3**{*y*} and slightly colored chalcones **1**{*x,y*}. The filtration of the crude reaction product through a silica gel plug, introduced in this work, allowed the complete removal of the Ph_3_P=O affording highly pure chalcones with isolated yields in the range of 80–100%, better in general than those obtained using the aldol condensation. Furthermore, the Wittig reaction allows the use of an excess of the ylide **9**{*x*} because it is retained in the silica gel plug together with the Ph_3_P=O. The use in the aldol condensation of an excess of the corresponding acetophenone **2**{*x*} is not adequate because in many cases such excess co-eludes with the final chalcone **1**{*x,y*} in the column chromatography required. A recrystallization from EtOH is an improvement but often causes a considerable reduction in yield.

The only drawback of the Wittig protocol is the lack of a broad commercial offering of ylides **9**{*x*} with a variety of ring substituents. However, these ylides can be obtained in two steps from 2-bromoacetophenones **10**{*x*}.

Consequently, we recommend the use of the Wittig protocol, including the silica gel plug filtration, as a general method for the synthesis of chalcones **1**{*x,y*} in high yield and excellent purity. Even though we have not tested the aforementioned Wittig protocol with heterocyclic ylides or heterocyclic aldehydes, there is no indication that this protocol would not be better than the corresponding aldol condensation.

## 4. Materials and Methods

All solvents and chemicals were reagent grade. Unless otherwise mentioned, all solvents and chemicals were purchased from commercial vendors (Sigma Aldrich (Burlington, MA, USA), ABCR (Karlsruhe, Germany), Fluorochem (Hadfield, UK), Apollo Scientific (Stockport, UK), Activate Scientific (Prien, Germany), Alfa Aesar (Ward Hill, MA, USA), and ACROS Organics (Antwerp, Belgium)) and used without further purification.

^1^H- and ^13^C-NMR spectra were recorded on a Varian 400-MR spectrometer (^1^H NMR at 400 MHz, and ^13^C NMR at 100.5 MHz). Chemical shifts were reported in parts per million (δ) and are referenced to the residual signal of the solvent DMSO-*d*_6_ 2.50 ppm or tetramethylsilane (TMS) 0 ppm in ^1^H NMR spectra and the residual signal of the solvent DMSO-*d*_6_ 39.5 ppm in ^13^C NMR.

General protocol for the Wittig synthesis of chalcones **1**{*x,y*}:

An amount of 1.5 mmol of the corresponding ylide **9**{*x*} is suspended in 5 mL of distilled water and 1.0 mmol of the corresponding benzaldehyde **3**{*y*} is then added. The solution is stirred at reflux temperature until complete (monitored by TLC using 100% DCM as eluent and ^1^H-NMR). The resulting solution is then cooled down and extracted with 3 × 10 mL of DCM. The combined extracts are dried with anhydrous MgSO_4_ and concentrated in vacuo to around 2–5 mL The resulting solution is filtered through a silica gel plug (silica gel 60, 0.063–0.200 mm, Millipore 1.07734, 1.5–2 cm of height on a 2 cm diameter filtering plate) and washed with DCM until it comes out uncoloured (100–200 mL depending on the case, a TLC in DCM can be used to establish the final point of the elution). Finally, the solvent is concentrated in vacuo to afford the corresponding chalcone **1**{*x,y*} that is analysed using ^1^H- and ^13^C-NMR.

General Aldol protocol for the synthesis of chalcones **1**{*x,y*}:

An amount of 3.23 mmol of the corresponding benzaldehyde **3**{*y*} is dissolved in 7 mL of EtOH followed by 3.26 mmol of the corresponding acetophenone **2**{*x*} and 0.391 mmol of KOH. The mixture is heated at 40 °C in an ultrasound bath until reaction completion (monitored by ^1^H-NMR). The solvent is then removed under reduced pressure and the resulting solid (in some cases an oil) is analysed ^1^H- and ^13^C-NMR. The crude resulting chalcone **1**{*x,y*} is recrystallized from EtOH or purified by column chromatography.

The chalcones **1**{*x,y*} obtained in this work were previously described in the literature and the spectra obtained for them are in agreement with the literature data reported respectively in SDBSWeb (https://sdbs.db.aist.go.jp, accessed on 10 October 2023, National Institute of Advanced Industrial Science and Technology) or in a publication as follows: **1**{*1,1*} (G = H, SDBS-No: 5818), **1**{*1,2*} (G = *p*-NMe_2_, SDBS-No: 11614), **1**{*1,3*} (G = *p*-Me) [33], **1**{*1,4*} (G = *p*-Ome, SDBS-No: 11610), **1**{*1,5*} (G = *p*-F) [34], **1**{*1,6*} (G = *p*-Br, SDBS-No: 11601), **1**{*1,7*} (G = *p*-NO_2_, SDBS-No: 5679), **1**{*1,8*} (G = *p*-CN, SDBS-No: 11654), **1**{*1,9*} (G = *p*-COOMe) [35], **1**{*1,10*} (G = *o*-Me) [36], **1**{*1,11*} (G = *o*-F) [34], **1**{*1,12*} (G = *o*-Br) [37], **1**{*2,1*} (R = *p*-Me) [38], **1**{*3,1*} (R = *p*-NO_2_, SDBS-No: 6385).

## Figures and Tables

**Table 1:** Comparison between aldol condensation and Wittig protocols for the synthesis of chalcones **1**{*1,y*} from *para*-substituted benzaldehydes **3**{*2*–*9*} and acetophenone **2**{*1*} or ylide **9**{*1*}.

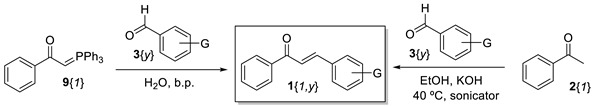					
Aldehyde	Chalcone	Wittig Reaction		Aldol Condensation	
		Reaction Time	Yield (%) ^1^	Reaction Time	Yield (%) ^2^
**3**{*2*} (G = *p*-NMe_2_)	**1**{*1,2*}	68 h	93	2.5 h	58 ^3^
**3**{*3*} (G = *p*-Me)	**1**{*1,3*}	19 h	91	2.5 h	74 ^3^
**3**{*4*} (G = *p*-OMe)	**1**{*1,4*}	21 h	96	2.5 h	54 ^3^
**3**{*5*} (G = *p*-F)	**1**{*1,5*}	45 min	95	2.5 h	80 ^3,4^
**3**{*6*} (G = *p*-Br)	**1**{*1,6*}	30 min	97	40 min	79 ^3^
**3**{*7*} (G = *p*-NO_2_)	**1**{*1,7*}	30 min	97	35 min	100 ^3^
**3**{*8*} (G = *p*-CN)	**1**{*1,8*}	45 min	90	20 min	100 ^3^
**3**{*9*} (G = *p*-COOMe)	**1**{*1,9*}	1 h	90	75 min	100 ^3^

^1^ Conversions are always 100%. Products obtained by evaporation of the organic CH_2_Cl_2_ solution. ^2^ Products were isolated by evaporation of the EtOH solution. ^3^ Purified by recrystallization from EtOH. ^4^ Aldol condensation carried out at room temp.

**Table 2:** Comparison between aldol condensation and Wittig protocols for the synthesis of chalcones **1**{*1,y*} from *ortho*-substituted benzaldehydes **3**{*10–12*}.

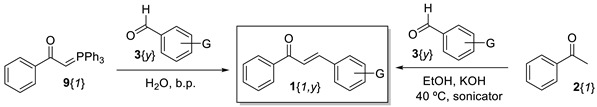					
Aldehyde	Chalcone	Wittig Reaction		Aldol Condensation	
		Reaction Time	Yield (%) ^1^	Reaction Time	Yield (%) ^2^
**3**{*10*} (G = *o*-Me)	**1**{*1,10*}	45 min	81	1 h	62 ^3^
**3**{*11*} (G = *o*-F)	**1**{*1,11*}	30 min	83	30 min	72 ^3^
**3**{*12*} (G = *o*-Br)	**1**{*1,12*}	30 min	83	12 h	65 ^4^

^1^ Conversions are always 100%. Products obtained by evaporation of the organic CH_2_Cl_2_ solution. ^2^ Products were isolated by evaporation of the EtOH solution. ^3^ Purified by recrystallization from EtOH. ^4^ Purified by column chromatography.

**Table 3:** Comparison between aldol condensation and Wittig protocols for the synthesis of chalcones **1**{*x,y*} from substituted acetophenones **2**{*2–3*} or the corresponding ylides **9**{*2–3*}.

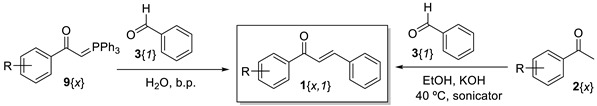					
Acetophenone or Ylide	Chalcone	Wittig Reaction		Aldol Condensation	
		Reaction Time	Yield (%) ^1^	Reaction Time	Yield (%) ^2^
**2**{*2*} or **9**{*2*} (R = *p*-Me)	**1**{*2,1*}	3.5 h	90	2 h	70 ^3^
**2**{*3*} or **9**{*3*} (G = *p*-NO_2_)	**1**{*3,1*}	3.5 h	73	2 h	50 ^3^

^1^ Conversions are always 100%. Products obtained by evaporation of the organic CH_2_Cl_2_ solution. ^2^ Products were isolated by evaporation of the EtOH solution. ^3^ Purified by recrystallization from EtOH.

## Data Availability

Data are contained within the article.
